# The influences of four types of soil on the growth, physiological and biochemical characteristics of *Lycoris aurea* (L’ Her.) Herb

**DOI:** 10.1038/srep43284

**Published:** 2017-02-27

**Authors:** Miaohua Quan, Juan Liang

**Affiliations:** 1 College of Biological and Food Engineering, Huaihua University, Huaihua, Hunan 418008, P. R. China; 2 Key Laboratory of Hunan Province for Study and Utilization of Ethnic Medicinal Plant Resources, Huaihua, Hunan 418008, P. R. China; 3 Key Laboratory of Hunan Higher Education for Hunan-western Medicinal Plant and Ethnobotany, Huaihua, Hunan 418008, P. R. China

## Abstract

Based on the characteristics of *Lycoris aurea (L. aurea*) natural distribution and local soil types, we selected four representative types of soil, including humus soil, sandy soil, garden soil and yellow-brown soil, for conducting the cultivation experiments to investigate key soil factors influencing its growth and development and to select the soil types suitable for cultivating it. We found that there existed significant differences in the contents of mineral elements and the activities of soil enzymes (urease, phosphatase, sucrase and catalase) etc. Among which, the contents of organic matters, alkali-hydrolysable nitrogen, Ca and Mg as well as the activities of soil enzymes in humus soil were the highest ones. In yellow-brown soil, except for Fe, the values of all the other items were the lowest ones. Net photosynthetic rate (*P*_n_), biomass and lycorine content in humus soil were all the highest ones, which were increased by 31.02, 69.39 and 55.79%, respectively, as compared to those of yellow-brown soil. Stepwise multiple regression analysis and path analysis indicated that alkali-hydrolysable nitrogen, and Ca etc. were key soil factors influencing *P*_n_, biomass and lycorine content of *L. aurea*. Thus, humus soil can be used as medium suitable for artificial cultivation of *L. aurea*.

*Lycoris aurea* (L’ Her.) Herb (*L. aurea*), also known as *Golen Magic Lily*, is a perennial herbaceous plant belonging to the genus *Lycoris*. It is a traditional Chinese medicinal herb plant^1^. Its bulb is rich in more than 10 types of alkaloids, including lycorine, galanthamine and lycoramine etc. and can be used to treat several important diseases such as poliomyelitis sequel, Alzheimer’s disease, and myasthenia Gravis etc. It also possesses certain anti-cancer effects and has been used in treating cancer. Thus, it has important medicinal value^2^. Lycorine belongs to pyrrolo-phenanthridine alkaloid within the class of isoquinoline alkaloids and is one of the major components of the anti-cancer alkaloids present in the plants in the family *Amaryllidaceae*^3^,^4^. Moreover, *L. aurea* is also a good groundcover and ornamental flower plant. Its bulb is also rich in starch and galanthus nivalis agglutinin. Thus, it is valuable to be widely applied in many fields, including landscape garden, industry and agriculture^5^. Its bulb contains many types of abundant components such as alkaloids and has relatively higher ornamental value. Thus, there is increasing market demand on *L. aurea*. However, in the recent years, the deterioration of the ecological environment and the over-artificial digging had led to the shortage of the resources of wild *L. aurea*. Thus, to initiate the artificial cultivation of *L. aurea* is of theoretical importance and practical significance for protection and proper utilization of the rare resource of wild *L. aurea*.

The quality of herb medicines is the comprehensive indicator reflecting certain cultivation technologies and ecological conditions under which the medicinal plants grow. Among which, soil serves as an essential medium for supporting plant growth and development, and thus, it has important influences on the growth, development and the medicinal quality of herb plants^6^. The nutritional elements (e.g. N, P, K, Ca, and Mg etc.) of soil are required for the growth of medicinal plants. These elements are not only the important sources of materials for building up the structures of plant tissues, but also are actively involved in the metabolic activities within plants^7^. For instance, Barlóg^8^ reported that magnesium and nitrogenous fertilizers were favorable for the growth, the biosynthesis and accumulation of alkaloids in *Lupinus angustifolius*. Ca^2+^ plays important roles in sequestration and signaling in regulating the activities of chloroplasts^9^,^10^. Plants require K^+^ for important intracellular physiological functions, including photosynthesis and nutrient transport^11^. Soil enzymes are one type of the most important biological components of the soil ecosystem. They play an important role in organic matter decomposition and nutrient cycling^12^. For instance, the hydroxylases (e.g. urease and sucrase etc.) can hydrolyze the macromolecules, such as proteins and polysaccharides, to form the simpler and smaller molecules that are easily absorbed by plants and to accelerate the nitrogen cycle and carbon cycle within the soil ecosystem. The activities of soil enzymes are closely related to soil physicochemical properties, soil types, and fertilizer application, cultivation and other agricultural measures^13^,^14^. Alkaloids are an important class of plant secondary metabolites and the result of the interactions between plants and their environments (both biotic and abiotic) during the long-term evolution process^15^,^16^. Different types of soil possess different textures and physiochemical properties while the demands of different types of medicinal herb plants for suitable soil conditions are quite different. Thus, the types of soil for cultivation of medicinal herb plants should be selected according to the particular physiological requirements of the particular plants^6^,^17^. Currently, most of the studies on *L. aurea* have mainly focused on the such aspects as biological evolution^18^,^19^, chemical compositions^20^,^21^,^22^, physiology and biochemistry^23^,^24^,^25^, pharmacology and pharmacodynamics^2^,^26^,^27^. In term of cultivation, Zeng *et al*.^28^ reported that *L. aurea* preferred the environments of shading, humidity, pleasantly cool, ventilation and penetrating light and had no strict requirement for soil type, but it grew better in sandy loam and calcific soil etc. that were fertile, porous, and rich in organic matter. However, the studies on the effects of soil conditions on the growth and development of *L. aurea* and the accumulation of the medicinal components have been barely available. Thus, it is necessary to select suitable soil conditions for artificial cultivation of *L. aurea*. In this study, based on the characteristics of its natural distribution patterns, we selected four representative types of soil with different textures and physiochemical properties for conducting the controlled experiments on the cultivation of *L. aurea,* aiming to study the correlations of the key soil factors with its growth, development and accumulation of medicinal component for providing the experimental basis for artificial cultivation of *L. aurea.*

## Results

### Comparison in physicochemical properties among different types of cultivation soil

As shown in Table 1, there were significant differences in pH value, the contents of soil moisture, organic matter, alkali-hydrolysable nitrogen, rapidly available phosphorus, rapidly available kalium, Ca and Mg etc. in different types of soil. Among which, the contents of organic matter, alkali-hydrolysable nitrogen, Ca and Mg were the richest ones in humus soil, which were 22.64, 7.99, 11.66 and 5.88 times those of yellow-brown soil, the poorest ones. The Fe content in yellow-brown soil was the highest one. The contents of soil moisture, rapidly available phosphorus, rapidly available kalium, Zn, Mn and Cu in garden soil were higher but its Mo content was extremely low. The Mo content in sandy soil was the highest one while the contents of the remaining compositions were between the other types of soil. The humus soil, sandy soil and garden soil were all alkalescent while yellow-brown soil was acidic.

### Comparison and analysis on major agronomic trials of *L. aurea* among different types of cultivation soil

Different types of cultivation soil had different impacts on the major agronomic trials of *L. aurea* (Table 2). The biomass performances, the bulb diameter, floral axis height, leaf length and leaf width were all the highest ones in humus soil, which were increased by 69.39, 14.50, 11.74, 15.72 and 8.06%, respectively, as compared to those in yellow-brown soil in which their performances were poorest. The differences in these parameters between two types of soil were statistically significant (P < 0.05). Their performances in sandy soil and garden soil were between those of the humus soil and yellow-brown soil.

### Comparison and analysis on photosynthetic parameters of *L. aurea* among different types of cultivation soil

Different types of cultivation soil had different effects on photosynthetic parameters of *L. aurea* (Table 3). Among which, the net photosynthetic rate (*P*_n_), chlorophyll content, transpiration rate (*T*_r_), intercellular CO_2_ concentration (*C*_i_) and stomatal conductance (*G*_s_) were all the highest ones in humus soil. Except for the lowest *T*_r_ value in sandy soil, the performances of all the remaining parameters in yellow-brown soil were the poorest ones. Compared to those in yellow-brown soil, *P*_n_ and chlorophyll content were significantly increased by 31.02 and 25.32%, respectively (P < 0.01).

### Comparison and analysis on the activities of soil enzymes among different types of cultivation soil

As shown in Table 4, there existed differences, to certain extend, in the activities of soil enzymes of *L. aurea* among four types of cultivation soil. Among which, the activities of soil enzymes in humus soil were the highest ones whereas those in yellow-brown soil were the lowest ones. Compared to those in yellow-brown soil, the activities of urease, sucrase, phosphatase and catalase were significantly increased by 9.63, 1.64, 4.03 and 1.95 times, respectively (P < 0.01). Among the soil enzymes tested, the activities of both urease and sucrase in four types of soil were also higher whereas the activity of catalase was the lowest one.

### Comparison and analysis on the lycorine content of *L. aurea* among different types of cultivation soil

**Figure 1:**
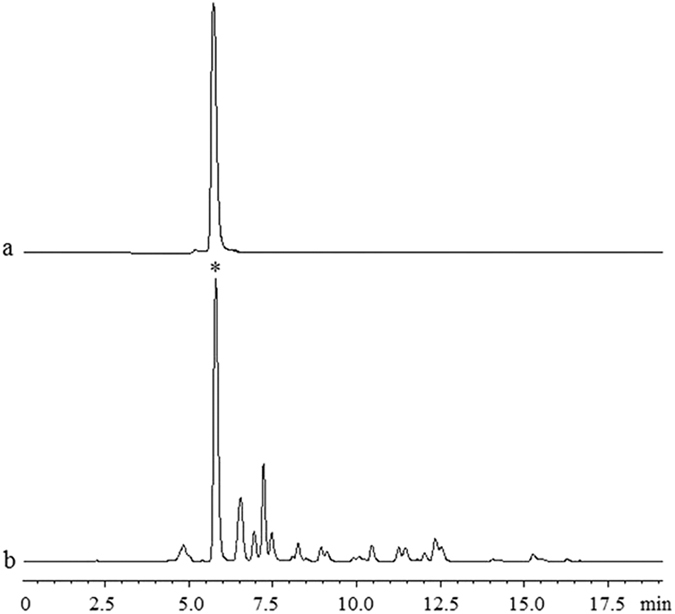
Chromatograms of lycorine reference substance (a) and bulb sample (b). *The objective peak of lycorine.

The chromatogram of the bulb sample of *L. aurea* in cultivation soil were shown in Fig. 1b. The lycorine content of *L. aurea* in four types of cultivation soil was in the order from high to low as follows: humus soil (1.48 mg g^−1^ DW) >sandy soil (1.35 mg g^−1^ DW) >garden soil (1.27 mg g^−1^ DW) >yellow-brown soil (0.95 mg g^−1^ DW). Among which, the lycorine content of *L. aurea* in humus soil was significantly increased by 55.79%, as compared to that in yellow-brown soil (P < 0.01).

### Analysis on key soil factors significantly influencing the medicinal quality of *L. aurea*

#### Stepwide multiple regression analysis on soil factors significantly influencing the medicinal quality of *L. aurea*

The measured values of the soil nutrients and mineral elements were taken as the soil factor group while the measured values of *P*_n_, biomass and lycorine content were taken as the *L. aurea* medicinal quality group. The soil factors, including pH (*X*_1_), soil moisture (*X*_2_), organic matter (*X*_3_), alkali-hydrolysable nitrogen (*X*_4_), rapidly available phosphorus (*X*_5_), rapidly available kalium (*X*_6_), Ca (*X*_7_), Mn (*X*_8_), Fe (*X*_9_), Na (*X*_10_), Zn (*X*_11_), Cu (*X*_12_), Mn (*X*_13_), and Mo (*X*_14_), were taken as the independent variables, i.e. soil factor group while the leaf *P*_n_ (*Y*_1_), biomass (*Y*_2_) and lycorine content (*Y*_3_) of *L. aurea* were taken as the dependent variables, i.e. medicinal quality group. The soil factors were selected with stepwide multiple regression method. The stepwide multiple regression equations between the *L. aurea* medicinal quality and significant soil factors were formulated (Table 5). As shown in Table 5, the significant soil factors influencing leaf *P*_n_ were alkali-hydrolysable nitrogen (*X*_4_) and Mo(*X*_14_) while the significant soil factor influencing the biomass was alkali-hydrolysable nitrogen (*X*_4_); the significant soil factors influencing the lycorine content were alkali-hydrolysable nitrogen (*X*_4_), Ca (*X*_7_) and soil moisture (*X*_2_). Thus, the significant soil factors influencing the medicinal quality of *L. aurea* are alkali-hydrolysable nitrogen, Ca, Mo and soil moisture.

#### Path analysis on the key soil factors significantly influencing *P*_n_, biomass and lycorine content of *L. aurea*

In order to further confirm the key soil factors significantly influencing *P*_n_, biomass and lycorine content of *L. aurea,* SPSS statistics analysis was conducted on the significant soil factors and the results were presented in Table 6. As shown in Table 6, the effects of alkali-hydrolysable nitrogen on *P*_n_, biomass and lycorine content of *L. aurea* were the greatest ones with determination coefficients of 0.927, 0.976 and 0.833, respectively, indicating that among these factors, alkali-hydrolysable nitrogen is the most significant one; The determination coefficient for the effect of Ca on lycorine content of *L. aurea* was 0.666, which was ranked the second place among the factors tested, indicating that Ca has important effect on the accumulation of lycorine in *L. aurea*. Mo had little direct effect on leaf *P*_n_ of *L. aurea (R*^2^ = 0.015); Soil moisture had negative effect on the lycorine content as its determination coefficient was negative, indicating that soil moisture is a limiting factor. Thus, the key soil factors significantly influencing *P*_n_, biomass and lycorine content of *L. aurea* were alkaline-hydrolysable nitrogen and Ca etc.

## Discussion

*L. aurea* has been used as a traditional Chinese medicinal herb plant to treat several important diseases. However, the increasing demand on *L. aurea* is contradictory to the limited supply source of wild *L. aurea* due to the deterioration of its inhabitant environment and over-artificial digging. One of the effective ways to resolve this contradiction is to artificially cultivate it in large-scale. Selection of appropriate types of soil for artificial cultivation of *L. aurea* is an essential step toward this solution. In the present study, we selected four representative types of soil, i.e. humus soil, sandy soil, garden soil and yellow-brown soil, to investigate their effects on the growth, development and accumulation of alkaloids of *L. aurea*. We determined that humus soil could be the suitable soil type for artificial cultivation of *L. aurea,* as supported by several lines of evidence as follows: (a) Humus soil contained the most abundant organic matter, alkali-hydrolysable nitrogen, Ca and Mg; (b) Humus soil displayed the best performances in several important agronomic trials, including biomass, the bulb diameter, floral axis height, leaf length and leaf width; (c) Humus soil contained the highest activities of soil enzymes including urease, sucrase, phosphatase and catalase; and (d) *L. aurea* grown in humus soil contained the highest content of lycorine, an important alkaloid. Furthermore, we also found that the key soil factors significantly influencing *P*_n_, biomass and lycorine content of *L. aurea* were alkali-hydrolysable nitrogen and Ca etc.

Different types of soil have quite different physicochemical and biological properties, which have substantial effects on the growth, development and the active constituents of medicinal plants^29^. Thus, different plants have different demands for appropriate type(s) of soil. For instance, Liu *et al*.^6^ reported that the types and texture of soil were closely related to the growth and development of medicinal plants and that loam soil was the relatively ideal type of soil for the cultivation of root/stem-types of medicinal plants. The results obtained from this study have indicated that the humus soil displays the best comprehensive performances in both agronomic trials and physiological and biochemical characteristics including *P*_n_, biomass and lycorine content of *L. aurea* grown among four different types of soil tested, followed by those of sandy soil, garden soil, and yellow-brown soil in the order from high to low. The differences in comprehensive performances are partially due to the significant differences in texture, pH value and organic matter etc. among the types of soil tested. In this study, measurement of the general physicochemical properties of cultivation soils revealed that humus soil was rich in organic matter content with its looser texture and better permeability. Humus soil, sandy soil and garden soil were all alkalescent while yellow-brown soil was acidic. The correlation analysis revealed that the soil pH value displayed a positive correlation with the lycorine content of *L. aurea* (Supplementary Information), indicating that the alkalescent soil is favorable for the accumulation of lycorine of *L. aurea*. This result was consistent with that obtained by Chao *et al*.^30^, who reported that the alkaline soil in North China was favorable for the accumulation of alkaloids while the acidic soil and yellow-brown soil in South China were unfavorable for the accumulation of alkaloids. Furthermore, path analysis indicated that value for the direct effect of soil moisture on the lycorine content was negative, implying that high soil moisture content may be unfavorable for the accumulation of alkaloids such as lycorine in *L. aurea*. This result was consistent with those obtained by El-Shazly *et al*.^16^ and Bustamante *et al*.^31^, who reported that drought environment could enhance the biosynthesis of plant alkaloids. The rich organic matter that was constantly decomposed in humus soil can provide the stable supply of nitrogen nutrients etc. for the growth of plants. The physicochemical properties of humus soil, i.e. rich organic matter, alkalescent pH value, the looser texture and thus, lower soil moisture, are favorable for the growth, biosynthesis and accumulation of alkaloids such as lycorine of *L. aurea*.

Deficiency or shortage of any of the nutritional elements (e.g. N, P, K, Ca, and Mg etc.) will certainly affect the normal growth and development as well as the internal and external qualities of plants^7^. Nitrogen is the most important element among all the nutritional elements required by plants^32^. For instance, the biosynthetic processes of alkaloids require nitrogen involvement. The increased, adequate or surplus nitrogen source was found to be favorable for the biosynthesis of alkaloids in *Larkspur*^33^. The present study indicated that the contents of alkaline-hydrolysable nitrogen and Ca in humus soil were higher than those in the poorest yellow-brown soil. Its biomass and the lycorine content were increased substantially. Stepwide multiple regression analysis and path analysis indicated that alkaline-hydrolysable nitrogen was the most important soil factor, and Ca is the secondary factor, implying that the higher contents of alkaline-hydrolysable nitrogen and Ca in humus soil are favorable not only for the growth and development of *L. aurea* but also for the accumulation of alkaloids including lycorine. These results also further confirm that nitrogen nutrient and Ca are the important environment factors stimulating plant growth and the biosynthesis of alkaloids. NO_3_-N and NH_4_-N are two major forms of nitrogen nutrients that are absorbed and utilized by plants. The effectiveness of these two forms of nitrogen element on the growth and development of plants are dependent on the types of plants, the concentrations of NO_3_-N and NH_4_-N and their ratio. The absorption, transport and assimilation during the metabolism processes and the effects on the growth, development and physiological processes are significantly different^34^. During the cultivation of crops, nitrogen nutrient and water supply are two very important controlling factors. Thus, how to maximize the effects of nitrogen nutrients, water and Ca^2+^ on stimulation of the growth, development, and the accumulation of alkaloids such as lycorine of *L. aurea* and the underlying regulatory mechanisms remain to be further investigated.

The microorganisms inhabiting in soil (e.g. bacteria and fungi etc.) play extremely important roles in the formation of soil fertility and the inter-conversion of plant nutrients and also affect the permeability of root cells and root metabolism. They can modify the root secretion and change the rhizosphere nutrients^35^,^36^. In the present study, we found that among four types of soil tested, there existed significant differences in the activities of soil enzymes, including urease and sucrase. Among four types of soil, the humus soil was rich in nutrients, such as organic matter, The activities of its soil enzymes were also higher. But in the poorest yellow-brown soil, the mean activities of soil enzymes were lower. The higher activities of these soil enzymes in the humus soil may be mainly attributed to the higher abundance and activities of soil microorganisms, likely due to the favorable soil conditions for their growth. The high activities of these soil enzymes and active soil microorganisms can continuously drive the degradation and mineralization of soil organic matter and provide the stable supply of nitrogen nutrients etc. for the growth of plants and thus, they are favorable for the growth, development and formation and accumulation of alkaloids, including lycorine of medicinal plants.

## Conclusion

In this study, we found that among four representative types of soil tested, the humus soil displayed the best comprehensive performances in terms of the agronomic, physiological and biochemical characteristics including *P*_n_, biomass and lycorine content of *L. aurea*, followed by those of sandy soil, garden soil, and yellow-brown soil in the order from high to low. This humus soil contained higher levels of organic matter, the activities of soil enzymes and mineral elements such as alkali-hydrolysable nitrogen, rapidly available phosphorus, and Ca etc. Its texture was looser and its permeability was quite good. Thus, this type of humus soil was suitable for artificial cultivation of *L. aurea*. Stepwise multiple regression analysis and path analysis indicated that the key soil factors significantly influencing *P*_n_, biomass and lycorine content of *L. aurea* were alkaline-hydrolysable nitrogen and Ca etc. Soil moisture was a limiting factor, implying that high soil moisture content may be unfavorable for the accumulation of lycorine of *L. aurea*. Our findings provide not only the guidance for conducting artificial cultivation of *L.aurea,* but also the methods for accumulation of alkaloids including lycorine of medicinal plants.

## Materials and Methods

### Materials and Cultivation Plots

The material of *Lycoris aurea* (L’Her.) Herb is an acclimated cultivar original from Huaihau, Hunan Province, China. Given that different medicinal plants have different requirements for soil types suitable for their growth and development, in this study, based on the characteristics of the natural distribution of *L. aurea*, we selected four representative soil types with quite different textures and physicochemical properties, i.e. humus soil (looser texture and rich in nutrition), sandy soil (loose texture), garden soil (moderate texture) and yellow-brown soil (dense texture). These four types of soil were collected from the original ecological environment in August 2012 and placed on the same experimental field under the same climate conditions for artificial cultivation of *L. aurea*. This experiment was conducted in Botanical Garden of Huaihua University, Hunan, China. The coordinates of geographical location are 110°01’ E, 27°35’ N, and the level above sea is 267 m. The climate in this location belongs to subtropical humid monsoon. The mean annual atmosphere temperature was 16.9 °C and the mean annual rainfall was 1358.6 mm. A number of bulbs with uniform size were selected and cultivated in the spacing (20 × 20 cm) in the experimental plots. The area of the plot was 1 m^2^. Each experiment was repeated three times. All the other conditions, such as water and light, were the same. The plots were managed with conventional management. The experimental period was from August 2012 to December 2015. This study aimed to investigate the effects of different soil types on the growth, development and accumulation of alkaloids of *L. aurea* for finding out the appropriate soil type(s) and for providing the reference basis for artificial culture of *L. aurea.*

## Experimental Methods

### Measurement of the general physicochemical properties of cultivation soil

#### Soil sampling and measurement of the contents of elements

The soil samples (0–20 cm depth) were collected from the experimental plots in December 2014. The samples were air-dried at room temperature. After passed through a 2 mm sieve, the soil samples were used for analysis. The contents of elements such as Ca, Mg, Fe and Mn etc. in the samples were determined with Inductively Coupled Plasma-Mass Spectrometry (Agilent7700, USA) in Hunan Food Test and Analysis Center, according to Agricultural Industry Standards or National Quality Standards NY/T 87–1988 and NY/T 296–1995 etc^37^.

#### Measurement of other factors of the soil samples

The contents of alkali-hydrolysable nitrogen, rapidly available phosphorus, rapidly available kalium were determined with diffusion method, NaHCO_3_ extraction-Mo-Sb colorimetric method, and NH_4_OAc extraction-flame spectrophotometry, respectively. The organic matters were determined with potassium dichromate oxidation heating method. The soil water content was measured with oven-drying methd^38^. The pH value was determined by using of PHS-3C precision acidity meter.

#### Measurement of major agronomic trails

Because *L. aurea* has the following characteristics: its flowers and leaves do not appear at the same time and it has summer dormancy. Its flowers blossom out in August, its leaf development starts in September and its vigorous growth stage is in December. Thus, the measurements of its agronomic trails in different types of cultivation soil were conducted in two stages. Its floral axis height was measured in August 2014 while its morphological parameters, including leaf length, leaf width and the bulb size were measured in December of the same year. The entire plants, including roots, leaves and bulb, were collected and dried by baking in oven to the constant weight and its biomass was weighted. The bulb samples were ground into powder with grinder (60 meshes) and stored under dry condition for subsequent analysis. Five healthy and strong plants were randomly selected from each sampling site and used as the measured subjects. Three repeat experiments were set.

#### Measurement of photosynthetic characteristics

Photosynthetic parameters, such as leaf *P*_n_, *T*_r_, *G*_s_, and *C*_i,_ and other physiological factors were measured by using of *Li-*6400 portable photosynthesis measurement system with a red-blue light source (*Li-cor*, USA) under saturating light of 1 000 μmol m^−2^ s^−1^. The net photosynthetic rates were measured at least 30 min after the attainment of the temperature^39^. Given that an “afternoon relaxation of photosynthesis” phenomenon exists in *L. aurea*, measurement of photosynthesis was conducted in morning time. The same positions of the leaves of five randomly selected *L. aurea* were used to measure *P*_n_ during 9:00–11:00 am in December 2014 when the plant was in nutrition period^24^. Immediately after that, the corresponding leaves were collected and extracted in 95% ethanol and measured by spectrophotometric method (DU-800 spectrophotometer, Beckman Coulter, Inc.) at the wavelengths 665 and 649 nm. Contents of chlorophyll *a* and *b* were calculated by using the method of Lichtenthaler^40^.

#### Assays of activities of soil enzymes

In the middle 10 days of each month from January to December 2013, the soil samples at 2 cm below the surface soil nearby the root system of *L.aurea* were collected from various sampling sites. The enzymatic activities in these soil samples were assayed with the method reported by Guan *et al*.^41^ as follows: Urease activity was assayed with indophenol blue colorimetric method and expressed as the amount (in mg) of NH_3_-N/g of dried soil produced within 24 h incubation at 37 °C. Sucrase activity was assayed with 3,5-dinitrosalicylic acid colorimetry and expressed as amount (in mg) of glucose produced within 24 h incubation at 37 °C. Phosphotase activity was assayed with alkaline phosphatase colorimetric method and expressed as the amount (in mg) of phenol produced/g of dried soil within 24 h incubation at 37 °C. Catalase activity was assayed with potassium permanganate titration method and expressed as the volume (in mL) of consumption of 0.1 N KMnO_4_/g of dried soil within 20 min incubation at 37 °C. The mean annual activity of each of these enzymes was calculated.

### Measurement of lycorine content

#### Conditions used in assays with high-performance liquid chromatography (HPLC)

HPLC was used to measure of lycorine content. The chromatographic conditions used were set as following: 20 μL of samples or standards were injected into the Agilent Eclipse XDB-C18 column at 25 °C and eluted with mobile phase of 0.1% phosphoric acid:methanol of 65:35 at flow rate of 1.0 mL/min. The detection wavelength was at 288 nm^24^.

#### Linear regression

Lycorine content was measured with a LC-20AT HPLC (Shimadzu, Japan). 20 μL of lycorine standard (HPLC ≥ 98%, National Institutes for Food and Drug Control) solutions at concentrations of 20.0, 40.0, 60.0, 80.0 and 100.0 μg·mL^−1^ were injected into the column and separated under the above conditions. The equation of linear regression was as follows: *y* = 20648x + 11934, *R*^2^ = 0.9996. The chromatogram of the lycorine reference substance was shown in Fig. 1a. Its retention time of the objective peak was 5.833 min.

#### Sample preparation and measurement of lycorine content

The sieved samples of the bulbs were dried at 65 °C to constant weight. The sample was extracted with Soxhlet method^24^. The sample solution was separated as described above and lycorine content was calculated using the peak area according to the linear regression equation.

#### Data analysis

Data analysis was performed with Statistical Product and Service Solutions(SPSS). The correlation analysis was conducted with Pearson correlation coefficient method. The key soil factors influencing *P*_n_, biomass and lycorine content of *L. aurea* were determined with multiple regression analysis^42^ and path analysis^43^,^44^. The multiple regression analysis was performed with a stepwide method to sequentially include variables in the model, using the following pre-established criteria: inclusion of a variable when its level of significance was <0.05 (p in <0.05), exclusion of a variable when its level of significance was >0.10. This method selects significant variables one by one, and every time a new variable is included, the rest of those previously selected are examined to check if any of them may be removed from the model. The significance of coefficients was evaluated by a t-test. A p-value < 0.05 was considered significant.

## Additional Information

**How to cite this article:** Quan, M.H. and Liang, J. The influences of four types of soil on the growth, physiological and biochemical characteristics of *Lycoris aurea* (L’ Her.) Herb. *Sci. Rep.* **7**, 43284; doi: 10.1038/srep43284 (2017).

**Publisher's note:** Springer Nature remains neutral with regard to jurisdictional claims in published maps and institutional affiliations.

## Supplementary Material

Supplementary Information

## Figures and Tables

**Table 1:** Physicochemical properties of different cultivar soil types.

Physicochemical properties	Humus soil	Sandy soil	Garden soil	Yellow-brown soil
pH	7.37 ± 0.02^b^	7.65 ± 0.03^ab^	7.88 ± 0.05^a^	5.16 ± 0.03^c^
Soil moisture(%, FW)	19.87 ± 0.31^a^	16.69 ± 0.22^b^	20.21 ± 0.27^a^	16.57 ± 0.15^b^
Organic matter (g kg^−1^ DW)	216.35 ± 12.87^a^	75.38 ± 5.45^b^	50.26 ± 3.33^c^	9.15 ± 1.06^d^
Alkali-hydrolysable nitrogen (mg kg^−1^ DW)	415.94 ± 21.41^a^	295.62 ± 15.73^b^	281.45 ± 11.70^b^	46.25 ± 3.47^c^
Rapidly available phosphorus (mg kg^−1^ DW)	144.16 ± 11.54^b^	92.17 ± 8.67^c^	157.80 ± 10.90^a^	20.43 ± 1.08^d^
Rapidly available kalium (mg kg^−1^ DW)	438.87 ± 17.09^a^	387.61 ± 15.68^b^	457.18 ± 18.81^a^	177.14 ± 10.23^c^
Ca(g kg^−1^ DW)	67.61 ± 5.91^a^	57.07 ± 4.59^b^	41.79 ± 3.95^c^	5.34 ± 0.79^d^
Mg(g kg^−1^ DW)	33.15 ± 2.51^a^	28.19 ± 2.48^b^	7.08 ± 0.64^c^	4.82 ± 0.32^d^
Fe(g kg^−1^ DW)	27.39 ± 1.69^c^	39.89 ± 2.94^b^	50.69 ± 3.87^a^	51.39 ± 3.92^a^
Na(mg kg^−1^ DW)	2567.53 ± 112.85^a^	2447.01 ± 101.58^a^	2252.82 ± 105.69^bc^	2213.35 ± 97.89^c^
Zn(mg kg^−1^ DW)	265.13 ± 15.59^c^	338.20 ± 21.50^b^	877.91 ± 32.93^a^	174.06 ± 10.35^d^
Cu(mg kg^−1^ DW)	126.00 ± 10.14^c^	140.41 ± 10.66^b^	407.90 ± 21.53^a^	76.99 ± 5.99^d^
Mn(mg kg^−1^ DW)	695.87 ± 28.77^b^	646.92 ± 19.65^bc^	777.31 ± 35.21^a^	559.43 ± 21.86^d^
Mo(mg kg^−1^ DW)	23.01 ± 1.26^b^	33.90 ± 1.81^a^	0.35 ± 0.02^d^	14.70 ± 1.07^c^

Data followed by different letters within the same line are significantly different (p < 0.05). Mean ± *SM* represents their standards of error, n = 5. DW represents dry weight.

**Table 2:** Comparison of main agronomic characters of *L. aurea* in four types of soil.

Soil types	Biomass (g · plant^−1^ DW)	Bulb diameter (cm)	Floral axis height (cm)	Leaf	
				Length (cm)	Width (cm)
Humus soil	17.43 ± 1.56^a^	3.79 ± 0.15^a^	64.7 ± 5.75^a^	44.9 ± 4.92^a^	2.68 ± 0.15^a^
Sandy soil	14.12 ± 1.24^b^	3.45 ± 0.12^b^	63.3 ± 5.14^a^	43.6 ± 3.85^a^	2.61 ± 0.12^a^
Garden soil	14.54 ± 1.26^b^	3.48 ± 0.13^b^	64.1 ± 6.01^a^	42.5 ± 4.08^a^	2.59 ± 0.10 ^a^
Yellow-brown soil	10.29 ± 1.05^c^	3.31 ± 0.10^c^	57.9 ± 4.89^b^	38.8 ± 4.12^b^	2.48 ± 0.11^b^

Data followed by different letters within the same column are significantly different (p < 0.05). Mean ± *SM* represents their standards of error, n = 15. DW represents dry weight.

**Table 3:** Comparison of photosynthetic parameters of *L. aurea* in different cultivar types of soil.

Soil types	*P*_n_ (μmol · m^−2^ · s^−1^)	Chl (a + b) (mg · g^−1^ FW)	*T*_r_ (mmol · m^−2^ · s^−1^)	*C*_i_ (molCO_2_ · mol^−1^)	*G*_s_ (mol · m^−2^ · s^−1^)
Humus soil	11.15 ± 1.53^a^	1.93 ± 0.13^a^	2.98 ± 0.24^a^	327 ± 21.25^a^	0.239 ± 0.05^a^
Sandy soil	10.82 ± 1.48^a^	1.81 ± 0.12^a^	2.48 ± 0.21^b^	288 ± 23.47^b^	0.205 ± 0.02^b^
Garden soil	10.89 ± 1.12^a^	1.83 ± 0.13^a^	2.55 ± 0.19^b^	307 ± 26.83^a^	0.224 ± 0.02^a^
Yellow-brown soil	8.51 ± 1.15^b^	1.54 ± 0.11^b^	2.51 ± 0.14^b^	275 ± 19.82^b^	0.135 ± 0.01^c^

Data followed by different letters within the same column are significantly different (*P* < 0.05). Mean ± *SM* represents their standards of error, n = 15. FW represents fresh weight.

**Table 4:** Soil enzyme activities of different cultivation soil types.

Soil types	Urease (mg NH_3_-N g^−1^ DW,24 h)	Sucrase (mg glucose g^−1^ DW,24 h)	Phosphatase (mg g^−1^ DW,24 h)	Catalase (mL g^−1^ DW,20 min)
Humus soil	16.26 ± 1.08^a^	7.64 ± 0.51^a^	1.58 ± 0.12^a^	0.56 ± 0.06^a^
Sandy soil	12.43 ± 0.95^b^	7.51 ± 0.61^a^	1.56 ± 0.15^a^	0.41 ± 0.05^b^
Garden soil	7.58 ± 0.41^c^	4.12 ± 0.29^b^	0.98 ± 0.10^b^	0.48 ± 0.03^ab^
Yellow-brown soil	1.53 ± 0.11^d^	2.89 ± 0.24^c^	0.31 ± 0.04 ^c^	0.19 ± 0.02^c^

Data followed by different letters within the same column are significantly different (p < 0.05). Mean ± *SM* represents their standards of error, n = 5. DW represents dry weight.

**Table 5:** Stepwide multiple regression analysis on significant soil factors influencing *P*_n_, biomass and lycorine content of *L. aurea*.

Index	Regression equation	*R*^2^	t-test	P-value
*P*_n_ (*Y*_1_)	*Y*_1_ = 8.962 + 0.004*X*_4_ + 0.025*X*_14_	0.967	52.184	0.001
Biomass (*Y*_2_)	*Y*_2_ = 9.228 + 0.019*X*_4_	0.964	15.123	0.004
Lycorine content(*Y*_3_)	*Y*_3_ = 0.956 + 0.001*X*_4_ + 0.004*X*_7_ − 0.004*X*_2_	0.987	345.234	0.002

**Table 6:** Path analysis on key soil factors significantiy influencing *P*_n_, biomass and lycorine content of *L. aurea.*

Dependent variable	Independent variable	Correlation coefficient	Path coefficient	*R*^2^
*P*_n_	Alkali-hydrolysable nitrogen	0.975	0.822	0.927
	Mo	0.152	0.243	0.015
Biomass	Alkali-hydrolysable nitrogen	0.988	0.988	0.976
Lycorine content	Soil moisture	0.565	−0.034	−0.040
	Alkali-hydrolysable nitrogen	0.994	0.600	0.833
	Ca	0.996	0.425	0.666

*R*^2^ represents coefficient of determination.
