# Supplementary material for: The influences of four types of soil on the growth, physiological and biochemical characteristics of Lycoris aurea (L’ Her.) Herb
Source: Sci Rep. 2017 Feb 27;7:43284. doi: 10.1038/srep43284 (PMC5327428; doi:10.1038/srep43284)
Supplement: Supplementary Information [file srep43284-s1.doc]

**The influences of four types of soil on the growth, physiological and**

**biochemical characteristics of *Lycoris aurea* (L’ Her.) Herb**

Miaohua Quan^1,2,3^,*, Juan Liang^1^

*Corresponding author

**Name:** Miaohua Quan

**Professional Title:** Professor

**Professional degree:** Master

**Affiliation:** ^1^College of Biological and Food Engineering, Huaihua University，Huaihua, Hunan 418008,

P. R. China. ^2^Key Laboratory of Hunan Province for [Study and Utilization](http://tg5a45.mail.163.com/a/j/js3/java_script:showjdsw('jd_t','j_')) of Ethnic Medicinal

Plant Resources, Huaihua, Hunan 418008, P. R. China. ^3^Key Laboratory of Hunan Higher

Education for Hunan-western Medicinal Plant and Ethnobotany, Huaihua, Hunan 418008, P. R.

China.

**Address:** College of Biological and Food Engineering, Huaihua University, No.612, Yingfeng

Road, Huaihua, Hunan 418008, P. R. China.

**Phone #:** +86-745-2851-037; Fax: +86-745-2851-305.

**Email Address:** hhqmh100@163.com

**Supplementary Information**

**Supplemental material: Correlations of soil factors with the medicinal quality of *Lycoris aurea.*** The measured values of the soil nutrients and mineral elements were taken as the soil factor group while the measured values of *P*_n_, biomass and the lycorine content were taken as the group of medicinal quality of *Lycoris aurea*(*L. aurea*), correlations of which were analyzed. Among all the measured values of soil factors, the alkali-hydrolysable nitrogen displayed a significantly positive correlation with the biomass of *L. aurea* (p<0.05). Alkali-hydrolysable nitrogen and Ca displayed extremely significantly positive correlations with lycorine content (p<0.01). Alkali-hydrolysable nitrogen, rapidly available potasium, and Ca displayed significantly positive correlation with *P*_n_（p<0.05）. Except for Fe which displayed significantly negative correlations with *P*_n_, biomass and lycorine content, all the other soil factors including pH value, organic matters, rapidly available phosphorus, Zn, Mg, Cu, Mn and Mo etc. displayed positive correlations with the medicinal quality of *L. aurea*. In addition, the correlation coefficients of the leaf *P*_n_ of *L. aurea* with its biomass and lycorine content were 0.945 and 0.968, respectively. Among which, *P*_n_ was significantly correlated with lycorine content (p<0.05). The biomass of *L. aurea* displayed significantly positive correlation with the lycorine content in bulb (p<0.05).

**Competing financial interests:** The authors declare no competing financial interests.
